# The use of schools for malaria surveillance and programme evaluation in Africa

**DOI:** 10.1186/1475-2875-8-231

**Published:** 2009-10-19

**Authors:** Simon Brooker, Jan H Kolaczinski, Carol W Gitonga, Abdisalan M Noor, Robert W Snow

**Affiliations:** 1 Malaria Public Health & Epidemiology Group, Centre for Geographic Medicine Research - Coast, Kenya Medical Research Institute-Wellcome Trust Research Programme, Nairobi, Kenya; 2 Department of Infectious and Tropical Diseases, London School of Hygiene and Tropical Medicine, UK; 3 Malaria Consortium - Africa Regional Office, Kampala, Uganda; 4 Centre for Tropical Medicine, Nuffield Department of Clinical Medicine, University of Oxford, CCVTM, Oxford, UK

## Abstract

Effective malaria control requires information on both the geographical distribution of malaria risk and the effectiveness of malaria interventions. The current standard for estimating malaria infection and impact indicators are household cluster surveys, but their complexity and expense preclude frequent and decentralized monitoring. This paper reviews the historical experience and current rationale for the use of schools and school children as a complementary, inexpensive framework for planning, monitoring and evaluating malaria control in Africa. Consideration is given to (i) the selection of schools; (ii) diagnosis of infection in schools; (iii) the representativeness of schools as a proxy of the communities they serve; and (iv) the increasing need to evaluate interventions delivered through schools. Finally, areas requiring further investigation are highlighted.

## Background

The burden of malaria in some areas of sub-Saharan Africa (SSA) has started to decline over recent years: analyses of hospital admission data provide evidence of declining morbidity and mortality in Kenya [1,2], The Gambia [3], South Africa [4], Zanzibar [5], and Eritrea [6]. Such reductions have been variously attributed to the expanded distribution of insecticide-treated nets (ITNs), changing first-line treatments to artemisinin combination therapy (ACT) and increasing access to it, and the renewed use of indoor residual spraying (IRS). There are, however, fewer reports on the impact of these interventions on malaria transmission [5,7]. This is partly due to the technical and ethical difficulties associated with quantifying transmission using vector-based indices, such as the entomological inoculation rate [8]. A more frequently used malariometric index is the *Plasmodium falciparum *parasite rate (*Pf*PR): the proportion of surveyed persons harbouring parasites in their peripheral blood. The *Pf*PR among children aged 2-10 years provides an indirect quantitative measure of transmission intensity across a range of malaria endemicities [9,10]. Historically, the measurement of *Pf*PR had important roles during the first phase of the Global Malaria Elimination Programme (GMEP) and was subsequently used to monitor progress and verify interruption of transmission [11]. Moreover, contemporary maps of *Pf*PR can provide important information for national malaria control programmes by targeting interventions according to endemicity, thus cost-effectively targeting resources for malaria control [12,13].

Currently, the most robust sampling framework for national malaria surveys are household cluster surveys, including: Demographic and Health Surveys [14], the Multiple Indicator Cluster Surveys [15], and Malaria Indicator Surveys (MIS) [16]. All of these surveys collect household-level information on malaria intervention coverage, patterns of anti-malarial use, and in selected MIS, on the prevalence of malaria infection and anaemia among pregnant women and children under five years of age. However, estimating *Pf*PR among these age groups is not optimal as pregnant women sequester infections [17] and infection prevalence in very young children is modified by a variety of factors including presence of maternal antibodies [18,19]. More importantly, national cluster surveys are expensive, time-consuming, technically complicated to undertake, and sampling is typically powered to provide only national or in some instances, first-level administrative unit (e.g. State or Province) representative estimates of malaria risk and intervention coverage. Such limitations preclude frequent monitoring and evaluation, beyond the first administrative level, hindering decentralized planning and allocation of resources for targeted control. Increasing the frequency of monitoring risk enables prompt feedback of intervention effectiveness, helping control programmes to adapt and improve control strategies. Across much of SSA, routine collection of malaria information at district levels currently focuses on passive case detection data on suspected malaria cases compiled by health facilities. However, these data are almost universally incomplete [20], lack diagnostic precision [21], and are rarely used for planning purposes. Because of these shortcomings, alternative sampling methods for monitoring malaria risk and intervention coverage are being explored, including household lot quality assurance sampling [22,23] and expanded programme of immunization (EPI) contact sampling [24,25]. This article reviews the historical experience and current rationale for the use of schools and school children as a complementary, inexpensive framework for malaria planning, monitoring and evaluation.

## The history of school malaria surveys

**Figure 1:**
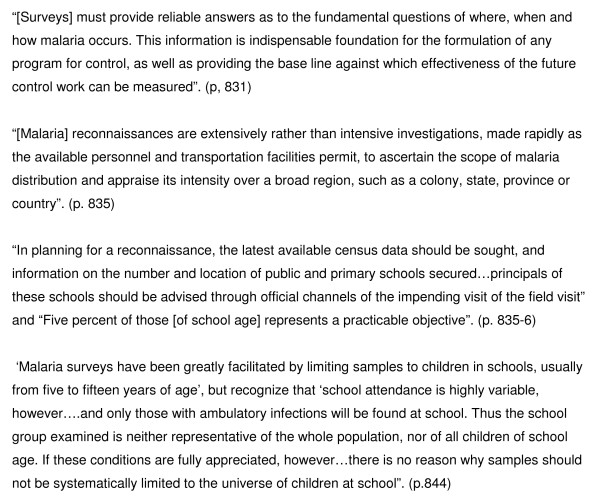
**Historical recommendations for planning and conducting school malaria parasite surveys **[26].

Conducting school malaria surveys is not a new approach in malariology. School surveys were a common feature of early approaches to malaria reconnaissance and control [26,27] (Figure 1). During the 1920-40s, large-scale school parasite surveys were frequently conducted in the United States and used to track the decline of malaria in the country: between 1942-43, for example, blood films were collected from 104,613 school children in seventeen states, with only 201 were found to be infected [28]. In the interests of time, survey investigations involved the examination of splenomegaly, limiting the collection of blood smears to children with enlarged spleens and/or a random proportion of children without palpable spleens. Such a screening approach was for instance adopted in nationally representative school malaria surveys undertaken in Cuba, 1935-1942 [29] and El Salvador, 1938-1940 [30].

School surveys were also an important component of early, particularly colonial, malaria reconnaissance and monitoring in Africa. In his survey of malaria in Southern Rhodesia, Alves reports results of school surveys conducted between 1937 and 1948 [31], while in the seminal 1967 book *Malaria in Tanzania *by Clyde [32] many of the reports of parasite prevalence were based on surveys of school children. In Uganda, as part of the pre-eradication programme of the 1960s, the nationwide distribution of different *Plasmodium *species was mainly based on school surveys conducted in different ecological settings across the country [33]. In Kenya, a recent assembly of parasite prevalence data found that over 50% of surveys conducted between 1975 and 1989 were Ministry of Health surveys of school children, which previously were a routine activity of the Division of Vector Borne Diseases (Noor et al. in preparation). School surveys were also an important component of monitoring the impact of malaria control: for example, the impact on malaria and anaemia of IRS in the Taveta-Pare area of Kenya and Tanzania during 1954 and 1959 was evaluated, in part, through school surveys [34,35]. Seemingly, however, school surveys fell out of favour, presumably due to financing constraints and a shift in the goals of control programmes from malaria elimination to morbidity control. More recently, school parasite surveys have been included in rapid assessments of malaria in urban SSA [36].

## Advantages of school-based malaria surveillance

The practical advantages of sampling children at school are clear: identification and selection of individuals is simplified, compliance is high, and costs are reduced, since only a fraction of the population is examined. In addition, ministries of education are increasingly developing or upgrading national school databases using geographical information systems (GIS). This allows incorporation of information on schools location and enrolment into a single database, and the use of geo-statistical methods to model risks between schools and across unsampled schools.

**Figure 2:**
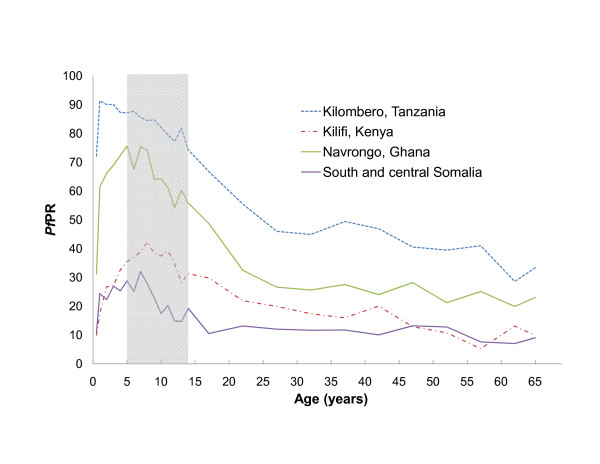
**The relationship between age and *Plasmodium falciparum *parasite rate (*Pf*PR) [Adapted from data provided in ref **[9]**]**. Each line represents the age profile for population living in varying transmission settings: South and Central Somalia (*Pf*PR among 5-14 year olds (*Pf*PR_5-14_) = 24.6%) [74]; Kilifi, coastal Kenya (*Pf*PR_5-14 _= 37.5%) [75]; Navrongo, Ghana (*Pf*PR_5-14 _= 66.9%) [76] and Kilombero, Tanzania (PfPR_5-14 _= 83.7%) [77]. Shaded box indicates typical age range of Primary school children in Africa (5-14 years).

There are additionally important epidemiological reasons for sampling children who attend school. Historically, malaria endemicity was defined on the basis of *Pf*PR among children aged 2-10 years and geographical reconnaissance of malaria was recommended in all areas prior to a control programme launching into an attack phase of the GMEP [11,33,34]. Recent mathematical models of malaria transmission dynamics indicate that EIR determines both the rate at which *Pf*PR rises during early childhood and the age at which maximum *Pf*PR is attained [9,10]. Age-stratified studies in varying transmission settings reveal that *Pf*PR consistency has a convex relationship with age, with *Pf*PR rapidly rising in among young children, attaining a maximum within the 5-10 year old age class, and declines among adolescents and young adults, thereafter maintaining a relatively stable low value throughout adulthood (Figure 2). Such consistency in the relationship between *Pf*PR and age settings permits age-standardization of available *Pf*PR estimates to the two to 10 year age range on the basis of catalytic conversion models [9], although estimates are most precise when the majority of the sampled population are within this age range.

## Monitoring intervention coverage

School-based surveys may potentially provide useful proximate estimates of the coverage of community-wide malaria interventions. Interviewing African school children by teachers is a well accepted and cost-effective approach for determining the prevalence of urinary schistosomiasis [37] and may potentially be used to monitor mosquito net coverage. Studies in Uganda show that schoolchildren reliably reported net ownership and the proportion of children protected in their households, hence providing a cheap and relatively fast method to estimate coverage at the community level [38,39]. The additional work involved in administering questionnaires to the pupils did not appear to pose any problems to the teachers, and importantly, school children were able to differentiate between treated and untreated nets [38]; though this will become less relevant as the use of long-lasting insecticide nets (LLINs) increases. The questionnaire approach may also be extended to monitoring household IRS coverage. By contrast, school children are unlikely to provide coverage information on the quality of disease management and treatment practices among their younger siblings or their pregnant mothers or sisters, and household surveys remain most relevant here.

## Selection of schools

The question as to how many schools to be included in malaria surveillance in a given area is similar to that faced by all large-scale surveys, and needs to be guided by both statistical and practical considerations. The appropriate sample size will depend on the expected *Pf*PR, the desired precision of *Pf*PR estimates, and the intrinsic variation of *Pf*PR between schools [40]. Practical constraints include availability of financial and human resources as well as accessible of schools.

**Figure 3:**
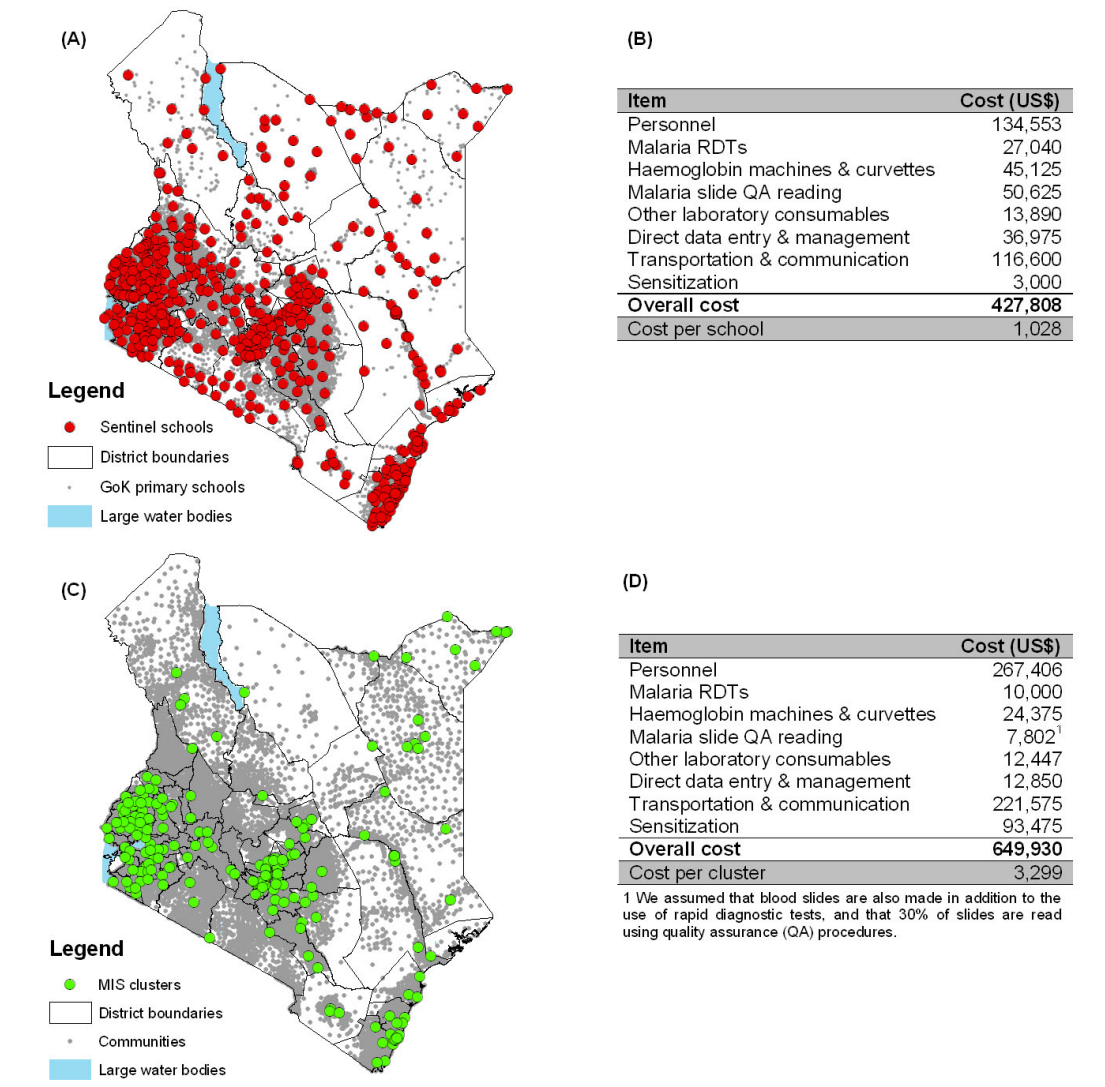
**Developing a national school-based malaria information system in Kenya, 2009. **(A) geographical location of the 416 sentinel schools in a national school malaria survey and all Government of Kenya (GoK) primary schools; and (B) the estimated personal, laboratory consumable, data management and logistics costs to implement a national school malaria survey in Kenya. (C) Geographical location of sampled 197 clusters in the 2007 Kenya Malaria Indicator Survey and all communities in Kenya; and (D) MIS costs, excluding training and piloting and technical support.

In traditional large-scale surveys, sampling units are typically selected on the basis of population proportionate sampling. Because there is need to define the geographical variation in *Pf*PR, a more spatially explicit sampling design may be required to capture spatial heterogeneities. Spatial sampling is an emerging area of scientific investigation [41,42], for which almost no work exists for parasite epidemiology. However, advances in GIS coupled with increasing availability of spatially-referenced national school databases provide an important opportunity for constructing efficient spatial sampling designs. In Kenya, for example, there are over 17,727 government, day mixed schools, which can constitute the sampling frame and these have all been recently geo-located using hand-held global positioning systems [43]. Schools can serve as the primary sampling unit and children within selected schools as secondary sampling units. By way of example, Figure 3a presents a representative national sample of 416 schools (3% of total number of schools) representing 41,600 children. Schools were selected proportionate to the number of schools in each district. The total estimated financial cost of such a survey is US$ 424,808, or US$ 1,021 per school surveyed (Figure 3b). In each school, 10 boys and 10 girls (plus two reserve children per school) from each class are selected using random number tables. By comparison, the nationwide MIS conducted in Kenya in 2007 was based on a two stage-stage cluster sample of 6,854 households surveyed in 199 census enumeration areas (Figure 3c), randomly selected from all regions and urban areas of the country. The survey included 33,534 persons, including 6,842 children aged 0-4 years. The corresponding survey costs excluding training, piloting and technical costs are estimated to be US$ 649,930, or US$ 3,299 per cluster sampled (Figure 3d).

School surveys may be further simplified by the use of lot quality assurance sampling (LQAS) [44], which has the potential to classify geographical areas according to (i) specified categories of infection prevalence to target interventions [45] or (ii) establish whether a specified coverage target has been reached [46]. Although LQAS has been extensively used to monitor coverage of vaccination programmes [23], there are few documented applications in malaria monitoring and surveillance. Current applications include community LQAS surveys for the detection of epidemics in the Madagascan highlands [45] and large country LQAS surveys of malaria intervention coverage as part of the World Bank's Malaria Booster Project in Nigeria [47]. Whatever the sample design, the usefulness of national school surveys will crucially depend on a number of issues, including the accuracy of diagnosis and how representativeness schools are of the wider community, which are now considered.

## Diagnosing infection in schools

The most common and widely used technique to detect *Plasmodium *sp. in circulating peripheral blood is microscopy. This method is, however, only as reliable as the quality of the blood slide preparation and equipment, skills of the microscopist and quality control [48,49]. An alternative and increasingly used diagnostic method are rapid diagnostic tests (RDTs). A number of products are now commercially available, detecting different parasite protein products: *P. falciparum*-specific histadine-rich protein (HRP) or species-specific isotypes of lactate dehydrogenase [50]. When stored and used correctly RDTs can have an accuracy that exceeds 95% sensitivity and 90% specificity for detection of *P. falciparum *when compared to expert microscopy even at relatively low parasite densities [51]. The tests with the highest sensitivity are typically HRP-based test since *Pf*-HRP can persist for two weeks after parasite clearance. Although *Pf*-HRP-based tests are more likely to generate false positives, which has implications on their use for case-management [52], these tests are still valuable during community prevalence surveys which aim to estimate parasite exposure as a measure of transmission intensity. The ease with which RDTs can be used and the short time in which they provide a diagnosis means that they can readily be incorporated into routine school malaria surveys, with identified positives treated on site. Recent experience in Kenya shows that the use of RDTs in 65 schools, compared to validated microscopy results, has a sensitivity of 98.9% and a specificity of 84.9% (Brooker et al. unpublished). A more cost-effective approach to improve precision is to use RDTs for field-based diagnosis in areas of low malaria transmission, with microscopy of all RDT-positive samples and a corresponding random selection of RDT-negatives undertaken by quality assured microscopists unaware of the RDT results. This stepwise approach to diagnosis has parallels with the two-stage approach of spleen examination and blood samples adopted in the USA, Cuba and El Salvador in the 1930s [29,30]. In areas of high transmission in Africa, the use of RDTs alone is advocated. Other diagnostic techniques are being explored to measure changes in infection exposure with time, notably serological measures of anti-malarial antibodies that persist for months or years after infection [53-56]. These approaches will become more valuable where school children may serve as sentinel surveillance to define malaria elimination progress in their communities.

## Representativeness of schools

**Figure 4:**
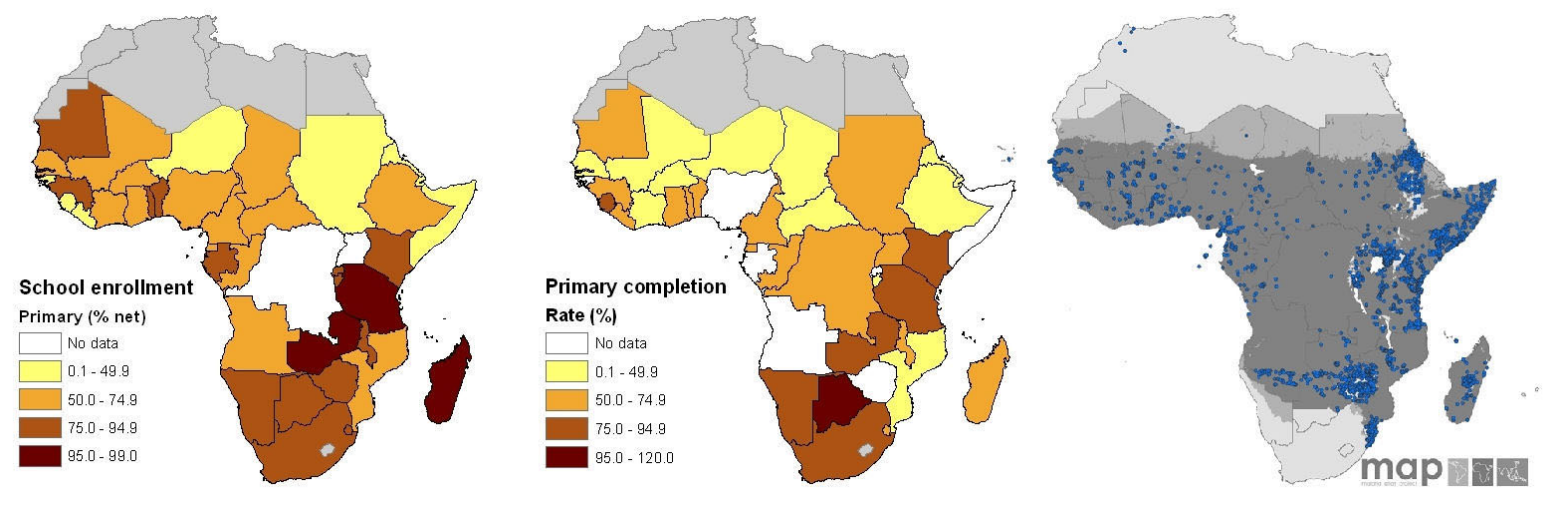
**Representativeness of school children for malaria surveillance in Africa**. (a) enrollment and (b) completion rates by country. Countries shaded grey indicate an absence of malaria transmission: Algeria, Egypt, Lesotho, Libya, Morocco, Tunisia and Western Sahara. Net enrollment ratio is the ratio of children of official school age who are enrolled in school to the population of the corresponding official school age. Primary completion rate is the percentage of students completing the last year of primary school. It is calculated by taking the total number of students in the last grade of primary school, minus the number of repeaters in that grade, divided by the total number of children of official graduation age. Data source: World Development Indicators [60]. (c) Geographical distribution of malaria survey data (n = 5307) currently included in the Malaria Atlas Project [63].

An important determinant of the representativeness of schools of the communities they serve is the level and equity of school enrollment. Current commitment of national governments and donors to achieving universal primary school participation [57], means that school enrollment rates are increasing. This has been most pronounced in SSA where since 2000 there has been a 52% increase in pupil enrollment [58]. Across the continent, however, there remains substantial variation in enrollment levels, and this will impact on the representativeness of schools. School enrollment in East Africa is characterized by high enrollment and primary completion rates [59]: for example, in Tanzania in 2007, net primary enrolment was 98% and primary completion rate 85% [60]. Evidence suggests that in such settings the majority of non-enrolled school age children have at least one sibling attending school, and that neither socio-economic status nor maternal education is associated with higher enrolment [59,61]. By contrast, in Central and West Africa, where both enrollment and completion rates are low, there are large differentials in enrollment by socio-economic and health status: for example, in Burkina Faso, only 18% from the poorest households enter grade 1 compared to 70% from the richest household [59]; and in Ghana, adolescent non-enrolled boys were more heavily infected with helminth infections and more likely to be anaemic than enrolled adolescent boys [62]. Such wealth differentials in enrollment have obvious implications for the schools representativeness of schools for the community at large where enrollment, infection and intervention use are affected by poverty. Representativeness will also depend on the types of schools sampled. In certain settings, boarding schools, single sex schools (such as Koranic schools), or non-government (private and parochial) schools may predominate; for example, parochial schools in Demographic Republic of Congo; and Koranic schools in Senegal. In such settings, it may be appropriate include non-government schools in the sampling frame. Representativeness of school surveys is likely to be greatest in those malaria endemic countries with the highest enrollment and completion rates, namely eastern and southern African countries (Figure 4). This will, in turn, help improve the coverage of Africa-wide empirical data to develop mapped extents of *P. falciparum *transmission intensity [63].

Finally, the representativeness of *Pf*PR data collected through school surveys may depend on size of the catchment area of schools. If, for example, a school's catchment area is large, it may include villages with varying malaria ecologies; if the catchment area is small, then the derived *Pf*PR is likely to be more representative of the immediate community. Little is known about the size and structure of school catchment areas in Africa, and this merits further investigation. It is known, however, that there is only a minor influence of household distance to schools on enrollment [64].

## Combing surveillance and intervention in schools

There is increasing recognition that universal mosquito net coverage is one of the most effective malaria prevention tools, such that LLINs should be distributed freely or should be highly subsidized and used by all community members, including school children [65]. In addition, school children themselves are increasingly becoming the targets of malaria control [66]. As the intensity of malaria transmission declines, it is suggested that clinical immunity will be acquired more slowly, with disease burdens shifting into older age groups, including school children [67,68]. This epidemiological transition is occurring at a time when more children than ever before are attending school. Consequently, there is growing interest in identifying malaria interventions that can be delivered through the existing school system [69], and control strategies for malaria in schools need to be formulated in relation to epidemiological patterns of infection and disease, as well as intervention cost-effectiveness. Monitoring and evaluation of school-based interventions will be essential to determine impacts of interventions on levels of infection, disease and school absenteeism. As such, schools become vehicles for surveillance of infection risks in the communities they serve and targets for intervention delivery. This too has a historical precedent: in Kenya, for example, during the 1970s and 1980s schools were used as a means to rapidly and routinely estimate infection risks across the country using technicians from regional offices of the Ministry of Health's Division of Vector Borne Diseases [70]. On completing these surveys staff provided chloroquine treatment for all infected children. Elsewhere in Africa, historical school-based delivery of malaria chemoprophylaxis was associated with significant reductions in malaria-related morbidity and mortality, and improvements in educational outcomes [71-73].

## Future directions

The routine surveillance of malaria infection is uncommon in any national malaria strategy in Africa and it is notable that countries in southern Africa have identified elimination as an achievable immediate target without any mapped reconnaissance of malaria risk. It is argued that surveys of school attending children provide a rapid, cheap and sustainable platform to assemble information on malaria risk among communities, and that in time, children attending school will increasingly become the focus of intervention as the epidemiology of malaria risk changes. This is not a new approach but its significance has re-emerged during a renewed international interest in malaria control and elimination. The use of schools as sentinels does however require further validation and optimization. Notable areas of investigation that require attention include studies on the representativeness of schools and how this varies in relation to levels of school enrolment as well as malaria endemicity; the potential sampling and diagnostic biases introduced when transmission intensity declines and all new infections become clinical events resulting in absenteeism; and the validity of children's reporting on intervention, especially mosquito net, use among household members. However, it is likely that as pupil enrollment continues to expand schools will increasingly provide a representative sample of community events. Furthermore, the technical capacity to conduct school malaria surveys already exists in most SSA countries, and engaging the health and education sectors in malaria control promotes inter-sectoral collaboration at national levels.

## Competing interests

The authors declare that they have no competing interests.

## Authors' contributions

SB and RWS conceived the idea for the paper, and SB developed the initial draft paper. JK and AMN contributed to the survey design issues, and CWG contributed to the issues surrounding the representativeness of schools. All authors read and approved the final manuscript.
